# Hemoglobin Levels and Red Blood Cells Distribution Width Highlights Glioblastoma Patients Subgroup With Improved Median Overall Survival

**DOI:** 10.3389/fonc.2020.00432

**Published:** 2020-04-17

**Authors:** Tehila Kaisman-Elbaz, Yonatan Elbaz, Vladimir Merkin, Lianne Dym, Ariel Noy, Maya Atar-Vardi, Romi Bari, Sivan Turiel, Adi Alt, Tali Zamed, Yael Eskira, Konstantin Lavrenkov, Yarden Kezerle, Victor Dyomin, Israel Melamed

**Affiliations:** ^1^Department of Neurosurgery, Soroka University Medical Center, Be'er Sheva, Israel; ^2^Faculty of Health Sciences, Ben-Gurion University of the Negev, Be'er Sheva, Israel; ^3^Physics Department, Nuclear Research Center - Negev, Be'er Sheva, Israel; ^4^Clinical Research Center, Soroka University Medical Center, Be'er Sheva, Israel; ^5^Institute of Oncology, Soroka University Medical Center, Be'er Sheva, Israel; ^6^Institute of Pathology, Soroka University Medical Center, Be'er Sheva, Israel

**Keywords:** glioblastoma multiforme (GBM), hemoglobin, RDW (red cell distribution width), prognostic factors, overall survival

## Abstract

Glioblastoma multiforme (GBM) is known for its dismal prognosis, though its dependence on patients' readily available RBCs parameters is not fully established. In this work, 170 GBM patients, diagnosed and treated in Soroka University Medical Center (SUMC) over the last 12 years were retrospectively inspected for their survival dependency on pre-operative RBCs parameters. Besides KPS and tumor resection supplemented by oncological treatment, age under 70 (HR = 0.4, 95% CI 0.24–0.65, *p* = 0.00073), low hemoglobin level (HR = 1.79, 95% CI 1.06–2.99, *p* = 0.031), and Red Cell Distribution Width (RDW) < 14% (HR = 0.57, 95% CI 0.37–0.88, *p* = 0.018) were found to be prognostic of patients' overall survival in multivariate analysis, accounting for a false discovery rate of < 5% due to multiple hypothesis testing. According to these results, a stratification tree was made, from which a favorable route highlighted a subgroup of nearly 30% of the cohorts' patients whose median overall survival was 21.1 months (95% CI 16.2–27.2)—higher than the established chemo-radiation standard first-line treatment regimen overall median survival average of about 15 months. The beneficial or detrimental effect of RBCs parameters on GBM prognosis and its possible causes is discussed.

## Key Points

– GBM resection followed by oncological treatment of patients under the age of 70 with normal hemoglobin level and RDW < 14% enhance patients' survival.
– Measures aimed to normalize hemoglobin levels and RDW prior to surgical intervention may be useful in order to improve GBM patients' prognosis.

## Introduction

Glioblastoma multiforme (GBM) is the most common primary malignant brain tumor in adults (1). Although scientific progress was made over the years, patients' overall survival patterns have not considerably changed. Due to the aggressive nature of GBM, maximal multi-modality treatment, given according to established chemo-radiation standard first-line treatment regimen (Stupps' protocol) (2), has managed to increase the median overall survival to approximately 15 months (3–5) but has not brought a cure to patients suffering from the disease.

GBM is an extremely heterogeneous, multifaceted disease that harbors different disciplinary parameters (i.e., clinical, radiological, molecular, laboratory features) whose combination eventually impacts the individual prognosis in a pattern not fully understood thus far. As of today, the prognostic significance of KPS (6), age (7), the extent of resection (8, 9), and selected molecular markers detection, i.e., MGMT methylation (10) and IDH 1/2 mutation (11, 12) are well-established. Other factors such as Ki67 (13), low CD4 lymphocyte count (14), hemoglobin level (15–19), Red Blood cells Distribution Width (RDW) (20, 21), and various anatomical features such as sub-ventricular zone involvement (22) were also shown to have prognostic value, but are still not as well-recognized as those previously mentioned.

Red Blood Cells (RBCs) parameters' association to cancer is well-known (23–25). It has been somewhat investigated previously with regard to GBM prognosis, e.g., Lutterbach et al. (16) pointed to low hemoglobin level as an adverse prognostic factor. This work was supported by subsequent works showing similar results (17, 18). Nevertheless, some works failed to demonstrate the significant prognostic value of hemoglobin level for GBM (18, 19, 26). Another parameter recently studied is RDW, which reflects a degree of variation (anisocytosis) in the size of the circulating red cells. A high RDW is associated with a significant increase in all-cause mortality rates (27) and could also predict poor overall survival of GBM patients (15, 20, 21, 28). Other RBCs parameters were even less studied in the context of GBM prognosis. Surprisingly, even with the standard availability of RBCs parameters, there is a limited number of papers concerning this topic, and the specific mechanism of influence was not thoroughly explored. Therefore, it is currently uncertain which features influence prognosis and what their relative impact on GBM overall survival is.

With the aim of identifying prognostic factors that influence the GBM disease course, a comprehensive database of 170 GBM patients treated in Soroka University Medical Center (SUMC) over the last 12 years was created. This paper presents retrospectively collected data from digital and paper archives with a focus on RBCs parameters, findings that were shown to affect patients' cohort prognosis.

## Methods

### Data Collection

In this retrospective study, the medical files of 170 pathologically verified GBM patients, treated in SUMC between the years 2006–2017 were reviewed, following institutional Helsinki committee approval. Data collection was finalized in October 2018. Demographics, diagnosis dates, procedure type, clinical course, imagery, laboratory, and histopathological data were retrieved and analyzed as described below. The main characteristics of the cohort are given in Table 1. Personal medical history was obtained with regard to documented chronic illnesses, for example, diabetes, hypertension, and preceding malignant diseases. Other medical conditions, such as pre-operative KPS, obesity, and anemia, were determined based on collected and analyzed data at the time of diagnosis. The surgical intervention type was determined as either biopsy or tumor resection. The extent of resection was not addressed in this data analysis. Adjuvant oncological treatments (i.e., radiation and chemotherapy) were regarded as given or not. Reviewing of patients' files, Ki67, which were constantly documented in the pathological report, was extracted. Routine IDH 1/2 mutation identification was established in SUMC only in 2017, and therefore, most patients included in this registry are with unknown IDH 1/2 mutation status. Pre-operative imagery data and tumor morphology were also gathered but the analysis of these parameters will be elaborated in a separate publication.

**Table 1:** Patients' cohort retrospectively collected main characteristics.

	**Variable**	**All**	**Treated**	**Partial/Non-treated**
*N*		170	112	58
Gender	Male	99	62	37
	Female	71	50	21
Age	Mean (±1σ)	62.4 (±14.9)	58.6 (±15)	69.8 (±11.6)
	<70 years	112	86	26
	≥70 years	58	26	32
KPS	Mean (±1σ)	76.2 (±13.1)	78.4 (±10.7)	71.9 (±16.1)
	≥70	147	103	44
	<70	23	9	14
Surgical intervention	Resection	132	112	20
	Biopsy	38	0	38
Oncological treatment	Full	127	112	15
	Partial/Non-treated	43	0	43
Chronic illnesses/Medical conditions	Diabetes	39	24	15
	Hypertension	74	43	31
	Obesity	37	26	11

*The entire cohort was divided into the treated group which includes patients whose tumor was resected followed by oncological treatment administration by the established chemo-radiation standard first-line treatment regimen (2) and a partially/non-treated group that includes patients who underwent suboptimal treatment other than that specified for the treated group*.

### Patients' Blood Panel

A complete blood count is routinely taken from each patient within 1–3 days prior to surgical intervention and automatically analyzed by ADVIA 2120 hematology system or Sysmex XN hematology analyzer. These laboratory values were obtained at constant time points and enabled us to link them to the patients' overall survival patterns with less bias. Pre-operative RBCs parameters examined included: hemoglobin, RBCs count, hematocrit (HCT), mean corpuscular hemoglobin (MCH), mean corpuscular hemoglobin concentration (MCHC), mean corpuscular volume (MCV), and RDW. Since the majority of patients were treated with steroids following hospital admission, and it has been well-established in the literature that steroids treatment can distort WBC and neutrophils count (29), WBC differential and related calculated ratios (e.g., neutrophil-lymphocytes ratio) were not addressed in this study. However, their effect on GBM survival has been addressed in recent years (30–33).

### Data and Statistical Analysis

Acquired data were analyzed using R^*^ software (34). Group-wise differences were assessed using Wilcox or Mann-Whitney *U*-tests. Survival analysis was done using univariate and multivariate Cox proportional hazard model, Kaplan-Meier overall survival curves, and log-rank test. When accounting for multiple hypothesis testing in retrospective study it is highly important to limit the type I errors. Hence the Benjamini-Hochberg (BH) procedure (35) accounting for dependency (36) as given by Benjamini-Hochberg-Yekutieli (BHY) was employed, in order to limit the expected value of the false discovery rate (FDR) to < 5%, and *p*-values were adjusted correspondingly (adjusted *p*-values are displayed within the tables). All average values in this study are provided, including their standard deviations (in parenthesis) and overall survival times are reported with their corresponding two-sided 95% confidence intervals.

### Overall Survival Analysis

In this work, the prognostic effects of various parameters on the patients' overall survival were examined. Overall survival was defined by the time between the surgical intervention (resection or biopsy), regarded as confirmed diagnosis time, until death by any cause or until the end of follow-up.

## Results

### Complete Cohort

Table 1 elaborates patients' cohort main characteristics. At the point of data analysis completion, 12 patients were alive and two were lost to follow, and their data were censored. Most patients diagnosed were males (58%) between the ages of 50–70 years (mean age of 62 years) which is consistent with other literature reports (1, 4, 37). The median overall survival of the entire patient cohort was 7.9 months (95% CI 6.7–10.6) (**Table 4**). Most patients (64%) did not survive longer than 12 months following diagnosis. Long-term overall survival was noticed in 6.5% (95% CI 3.5–12.5) and 3% (95% CI 1–8) of the patients who survived for 3- and 5-years, respectively (**Table 4**) which matches other reports (1). Patients' overall survival was compared between three diagnosis periods (years: 2006–2009, 2010–2013, 2014–2017). No statistically significant difference was detected between the groups (log-rank test *p* = 0.14), indicating that there is no treatment bias in the entire 12-year cohort, and hence the entire cohort can be further analyzed as homogenous. Past medical history and specific medical conditions of interest in diagnosis were also explored (e.g., hypertension, diabetes, obesity) and no remarkable effect on overall survival was found. Clinical presentation of most of the patients matched other studies (3) and included primarily focal motor impairment, confusion, seizures, and headaches.

### Treatment Effect Data Analysis

The entire 170 patients' cohort was subdivided into two groups: the treated group (*N* = 112), whose tumor was resected followed by oncological treatment administration by the established chemo-radiation standard first-line treatment regimen, and the partially/non-treated group (*N* = 58) that underwent suboptimal treatment other than that specified for the treated group (i.e., biopsy alone with or without partial oncological treatment). Subgroup characteristics are also displayed in Table 1. Importantly, the type of treatment decision was usually made considering age, functional status, co-morbidities, and a gross estimation of future ability to endure complimentary oncological treatments, as well as the will of the patient and family.

The treated group mean age was 58.6 years (±15 years standard deviation), with 84% of patients under the age of 70, while the partially/non-treated group mean age was 69.8 years (±11.6) with only 44% of patients under 70. The distribution difference passed a *t*-test with *p* < 0.0001. Preoperative KPS > 70 was recorded in 92% of treated patients [mean 78.4 (±10.7)], compared to 76% in the partially/non-treated group [mean 71.9 (±16.1)] (Table 1). This distribution difference also passed a *t*-test with *p* < 0.0001. This is in accordance with KPS and age being dominant factors in treatment modalities decision making.

The median overall survival of the treated group was 12.7 months (95% CI 10.7–15) compared to the partially/non-treated group, which was 3.1 months (95% CI 1.8–4.2) (**Table 4**). Partially/non-treated patients whose age was over 70, exhibit the poorest median overall survival−1.9 months (95% CI 1.5–3.4) compared to 3.4 months (95% CI 2.6–7.6) median overall survival of patients whose age was under 70. Univariate analysis showed that for the partially/non-treated group, only age under 70 was a marginally prognostic factor for overall survival (HR = 0.57, 95% CI 0.33–0.98, *p* = 0.042).

### Univariate and Multivariate Analysis of the Treated Group Overall Survival

It is well-established that several RBC parameters' normal range are gender-dependent (i.e., hemoglobin, RBC, HCT), though slightly different among worldwide institutions. Therefore, the cohort was divided by gender (Table 2), which by itself was not significantly correlated with patients' survival (Table 3). Nevertheless, the patients' hemoglobin, RBCs count, and HCT were significantly correlated with gender (*p* < 0.0001, *p* = 0.0007, and *p* = 0.0006, respectively), with higher average values demonstrated in males. MCH level was marginally significant (*p* = 0.035), with slightly higher levels in the male subgroup.

**Table 2:** Main variables of the treated group by gender distribution.

**Variable**	**Male (*N* = 62)**	**Female (*N* = 50)**	***p*-value**
Age [Years]	58 (±14.3)	59.4 (±15.9)	0.32
KPS	80 (±10)	77 (±11)	0.26
RBC [10^6^ cells/μL]	4.94 (±0.47)	4.63 (±0.46)	0.0007
HCT [%]	43.48 (±4.29)	40.71 (±3.43)	0.0006
Hb [g/dl]	14.65 (±1.44)	13.53 (±1.15)	<0.0001
MCV [fL/cell]	88.1 (±5.25)	88.3 (±4.74)	0.73
MCH [pg/cell]	33.72 (±1.18)	33.23 (±1.07)	0.035
MCHC [g/dL]	29.68 (±1.82)	29.35 (±1.7)	0.27
RDW [%]	13.6 (±0.94)	13.82 (±1.1)	0.3

*Age and KPS were not statistically significant. Several RBCs parameters in which cutoffs are known to be gender-dependent were shown to be higher in male than in female patients. Hb, hemoglobin; HCT, hematocrit; MCH, mean corpuscular hemoglobin; MCHC, mean corpuscular hemoglobin concentration; MCV, mean corpuscular volume; RDW, red blood cell distribution width*.

**Table 3:** Univariate and multivariate analysis of the 112 treated GBM patients' group.

		**Univariate**				**Multivariate**			
**Variable**	**Cutoff (*N* < / *N*>)**	**HR**	**95% CI**	***p*-value**	**Adjusted *p*-value**	**HR**	**95% CI**	***p*-value**	**Adjusted *p*-value**
Gender	F. vs. M. (50/62)	0.79	0.53–1.18	0.24	1				
Age (Years)	70 (86/26)	0.35	0.21–0.56	<0.0001	0.0029	0.4	0.24–0.65	0.00025	0.00073
KPS	70 (9/103)	2.12	1.01–4.43	0.047	0.23				
RBC	Low vs. normal (26/86)	1.82	1.16–2.87	0.01	0.073				
HCT	Low vs. normal (29/83)	1.8	1.13–2.88	0.014	0.082				
Hb	Low vs. normal (22/90)	2.3	1.39–3.8	0.0012	0.0117	1.79	1.06–2.99	0.031	0.031
MCV [fL/cell]	80 (10/102)	0.77	0.39–1.54	0.46	1				
MCH [pg/cell]	27 (9/103)	0.97	0.47–2	0.94	1				
MCHC [g/dL]	33 (41/71)	0.96	0.64–1.44	0.84	1				
RDW [%]	14 (75/37)	0.49	0.32–0.75	0.0011	0.0117	0.57	0.37–0.88	0.013	0.018

*Univariate analysis revealed age over 70, low RBCs count, low HCT, low Hb level, and RDW > 14% as unfavorable prognostic factors. P-values were adjusted following Benjamini-Hochberg and Benjamini-Yekutieli procedures to lower the expected value of the false discovery rate. Following these tests, only age under 70, normal Hb level, and RDW < 14% remained statistically significant following p-value adjustment. Multivariate analysis of those parameters also supplemented by the Benjamini-Hochberg, Benjamini-Yekutieli procedures, and p-values correction further demonstrated their statistical significance. Elaboration regarding cutoff selection is mentioned in the text. Hb, hemoglobin; HCT, hematocrit; MCH, mean corpuscular hemoglobin; MCHC, mean corpuscular hemoglobin concentration; MCV, mean corpuscular volume; RDW, red blood cell distribution width*.

In order to have a consistent gender-independent survival analysis taking into account the different gender average values of RBC, HCT and hemoglobin, a “Low” or “Normal” level for those three parameters was defined according to the SUMC laboratories reference values:

- RBC is “Low” when RBC < 4.2 × 10^6^ cells/mL in females or RBC < 4.7 × 10^6^ cells/mL in males and otherwise is “Normal.”
- HCT is “Low” when HCT < 37% in females or HCT < 42% in males and otherwise is “Normal.”
- Hemoglobin is “Low” when Hb < 12 g/dL for females or Hb < 14 g/dL for males and otherwise is “Normal.”

For all other RBCs parameters, cutoff values dividing the cohort between “Low” and “Normal” were determined based on the gender-independent accepted institutional cutoff, except for RDW, whose cutoff value was determined as 14% based on previous literature reports (15, 20, 33). The cutoff values are given in Table 3.

The univariate analysis using the Cox proportional hazard model is shown in Table 3. Three parameters were found to be prognostic with overall survival: (1) age under 70 (HR = 0.35, 95% CI 0.21–0.56, *p* ≤ 0.0001, adjusted *p* = 0.00029), (2) low hemoglobin level (HR = 2.3, 95% CI 1.39–3.8, *p* = 0.0012, adjusted *p* = 0.0117), and (3) RDW < 14% (HR = 0.49, 95% CI 0.32–0.75, *p* = 0.0011, adjusted *p* = 0.0117). Following the BHY procedure for FDR control application, all other parameters were not demonstrated as statistically significant factors for overall survival (Table 3). It is important to note that although KPS, RBC, and HCT passed the usual univariate analysis with *p* < 0.05, the BHY procedure excluded them as statistically significant prognostic parameters, highlighting the importance of strictly controlling for type I errors, even when the number of tested hypostases is not extremely large. In multivariate analysis, in age under 70 (HR = 0.4, 95% CI 0.24–0.56, *p* = 0.00025, adjusted *p* = 0.00073), low hemoglobin level (HR = 1.79, 95% CI 1.06–2.99, *p* = 0.031, adjusted *p* = 0.031), and RDW < 14% (HR = 0.57, 95% CI 0.37–0.88, *p* = 0.013, adjusted *p* = 0.018) remained significant as prognostic factors. Hence, among all parameters analyzed, age, hemoglobin level, RDW, and their combination as GBM prognostic factors were selected.

### Primary Stratification Tree and Overall Survival Curves

**Figure 1:**
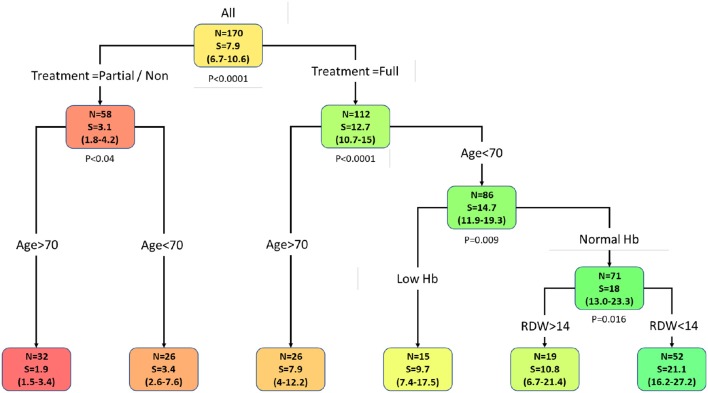
Primary stratification tree which presents cohorts' prognosis. Each colored box represents a specified subgroup with a number of patients (*N*), median overall survival in months (S), and 95% confidence interval (in brackets). The entire cohort was first divided into treated vs. partial/non-treated groups, which demonstrates a 4-fold increase in overall survival of the treated group compared to the partial/non-treated group. Notably, age under 70 is an independent favorable prognostic factor in both groups. Further stratification of treated patients whose age is under 70 revealed that normal hemoglobin level in those patients enhanced their overall survival to 18 months (95% CI 13–23.3) and RDW categorization to RDW < 14% highlighted a subgroup of 52 patients that survived for 21 months (95% CI 16.2–27.2). Hence, this cohort favorable prognosis group is characterized by the following four parameters: patients that underwent tumor resection supplemented with oncological treatment, age under 70, normal hemoglobin level, and RDW < 14%. Abbreviations: Hb, hemoglobin; RDW, red blood cell distribution width.

Using the data analysis presented above, an overall survival stratification tree was constructed and is shown in Figure 1. The complete survival data is given in Table 4. Each node in the tree lists the number of patients in the group, median overall survival in months, and its two-sided 95% CI. At each node split, a two-group log-rank test was performed and displayed. Focusing on the treated group, there are 86 patients whose age was under 70 and whose median overall survival was 14.7 months (95% CI 11.9–19.3) compared to 26 patients whose age was over 70 and whose median overall survival was 7.9 months (95% CI 4–12.2); none survived past the first year following GBM diagnosis. These results are consistent with the established GBM chemo-radiation standard first-line treatment regimen median overall survival rates (4, 5, 38).

**Table 4:** Median overall survival patterns of SUMC GBM patients by cohort group stratification.

**Model**	***N***	**Median overall survival**	**1 year [%]**	**2 year [%]**	**3 year [%]**	**5 year [%]**
ALL	170	7.9 (6.7–10.6)	36 (30–44)	15 (10–22)	6.5 (3.5–12.5)	3 (1–8)
Partial/Non-treated	58	3.1 (1.8–4.2)	8.5 (4–20)			
Age>70	32	1.9 (1.5–3.4)				
Age <70	26	3.4 (2.6–7.6)	15 (6–38)			
Treated	112	12.7 (10.7–15)	51 (43–61)	20 (13–29)	9 (5–17)	4 (2–12)
Age>70	26	7.9 (4–12.2)	23 (11–47)			
Age <70	86	14.7 (11.9–19.3)	59 (49–70)	25 (17–37)	12 (6–22)	6 (2–15)
Low Hb	15	9.7 (7.4–17.5)	37 (19–71)			
Normal Hb	71	18 (13–23.3)	63 (52–75)	31 (22–45)	15 (8–27)	8 (4–20)
RDW > 14%	19	10.8 (6.7–21.4)	33 (17–64)	11 (3–46)		
RDW < 14%	52	21.1 (16.2–27.2)	72 (61–85)	37 (26–54)	17 (9–32)	9 (3–23)

*The entire cohort demonstrates commonly accepted, including long-term, overall survival patterns. Importantly, patients included within the partial/non-treated group, regardless of their age, did not survive past the first year following GBM diagnosis. Similar overall survival patterns were demonstrated for treated patients whose age was over 70, or patients whose age was under 70 but with either low hemoglobin or RDW > 14%. Worst median overall survival was shown for patients whose age was over 70 years in the partial/non-treated group. In contrast, best median overall survival was demonstrated for patients in the treated group, whose age was under 70 years with a combination of normal hemoglobin level and RDW < 14%. Hb, hemoglobin; RDW, red blood cells width distribution*.

Stratifying patients whose age was under 70, those with normal hemoglobin level (*N* = 71) had an increased median overall survival of 18 months (95% CI 13.0–23.3). This patients' subgroup also demonstrated an enhanced overall long-term survival pattern with a 15% 3-year survival rate (95% CI 8–27). Further addition of RDW < 14% criteria for this group of patients showed an increased median overall survival of 21.1 months (95% CI 16.2–27.2). Therefore, an average increase of 6 months in the overall survival rate of treated patients whose age was under 70 was demonstrated when normal hemoglobin level was combined with RDW < 14%, with significant long-term survival rates of 17% (95% CI 9–32). This subgroup of patients consists of about 30% of all the patients in the entire cohort.

**Figure 2:**
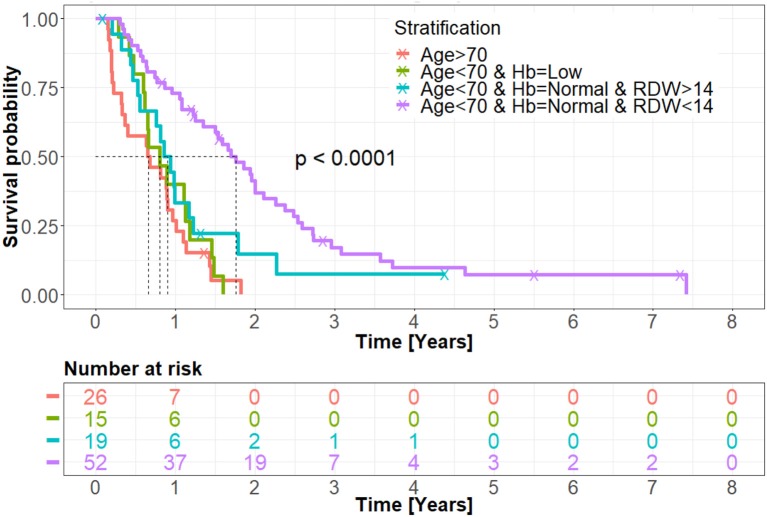
Kaplan-Meir overall survival curve of the treated group. Patients whose age was over 70 and patients whose age was under 70 but with low hemoglobin level demonstrated the worst prognosis of the entire treated cohort. According to the data analysis, these patients did not survive past the first year following GBM diagnosis. Patients whose age was under 70 with a normal hemoglobin level but with RDW > 14% showed a slight trend toward improved overall survival, though not statistically significant. Patients whose age was under 70, with normal hemoglobin level and RDW < 14%, exhibited the best cohort overall survival, with long-term survival patterns demonstrated. Hb, hemoglobin; RDW, red blood cell distribution width.

As demonstrated in the primary stratification tree, other routes are significantly unfavorable regarding patients' cohort prognosis. Figure 2 shows Kaplan-Meier overall survival curves calculated for the treated patients' group. Patients whose age was over 70 demonstrated the poorest overall survival. A slightly similar overall survival rate of patients whose age was under 70, with low hemoglobin level and patients whose age was under 70, with normal hemoglobin level and RDW > 14% was noticed. The most favorable survival pattern was demonstrated in patients whose age was under 70, with a normal hemoglobin level and RDW < 14% (*p* < 0.0001).

## Discussion

This study retrospectively analyzed routinely collected RBCs parameters of 170 newly diagnosed GBM patients to identify possible prognostic factors for their overall survival. The data analysis given in this work shows, for the first time, that both pre-operative normal hemoglobin levels and RDW < 14% are positive prognostic factors of GBM patients' survival whose age was under 70 and underwent tumor resection supplemented with oncological treatment. This group, consisting of about 30% of the entire cohort (52 out of 170 patients), exhibits a median overall survival of 21.1 months, which is significantly higher than the current median overall survival of GBM patients treated with the established chemo-radiation standard first-line treatment regimen (2).

The finding that low hemoglobin level is significantly associated with a lower survival rate of GBM patients is consistent with the known fact that tendency toward anemia is linked to lower survival rates in cancer patients (23, 25). In the context of GBM, several works supported low hemoglobin levels as a poor prognostic factor (16–19, 26), though other works were less supportive of this notion. Fiorentino and Fusco (26) studied the effect of hemoglobin level of mostly biopsied elderly GBM patients (median age of 71 years) and found that a hemoglobin level cutoff of ≥12 g/dL was not significantly related to overall survival but did show better progression-free survival. These results are in accordance to the study presented here, since patients whose age was over 70 were also mostly biopsied and did not show overall survival correlation with RBCs parameters checked. Céfaro et al. (18) examined high-grade gliomas population between the years 2001–2010 and found that hemoglobin level ≤12.0 g/dL was related to poor prognosis. Lutterbach et al. (16), Odrazka et al. (17), and Lally et al. (19) investigated hemoglobin level at cutoffs of 12–14 g/dL and showed a clear tendency of above cutoff hemoglobin levels being a positive prognostic factor. It is important to note that dividing GBM patients into two groups with a single hemoglobin cutoff may lead to misleading results since there is a well-established difference in the mean value of hemoglobin levels between genders. Also, these works were conducted before the established chemo-radiation standard first-line treatment regimen, adopted in 2005 (2). This makes it difficult to deduce from these findings the results obtained nowadays from studies including GBM patients treated with this protocol. Maas et al. (39) recently found no relation between pre-operative hemoglobin level and survival in 497 glioblastoma patients, though did not reproduced the already established dependency on age and KPS. A possible explanation for those results is the inclusion of partially treated and older age patients in the full multivariate analysis as opposed to the current work. A multivariate analysis on the current entire 170 patients' cohort also shows no significant prognostic effect of hemoglobin level, highlighting the importance of age and treatment stratification before analyzing RBCs parameters.

In addition to the hemoglobin level, RDW < 14% was shown to be a significant favorable prognostic factor for GBM overall survival. RDW, which also serves as an anemia marker, and its high values associated with a proinflammatory state, was recently linked to the decreased overall survival of cancer patients (lung, esophageal carcinoma, multiple myeloma, etc.) (24). Increased RDW [i.e., RDW ≥ 13.95% (15)] was negatively linked to glioma patients' disease course in several papers (15, 21, 27). But these results are not exclusive to GBM patients. Nevertheless, it was further supported by Liang et al. (20) who studied RDW in GBM patients and found that RDW ≥ 14.10% was a negative prognostic factor. Based on the data analysis presented in the current study, normal hemoglobin level, and RDW < 14% increase patients' overall survival 2-fold. This highlights the potential role of RBCs parameters in this GBM patients' cohort prognosis through a so far unknown mechanism that should be thoroughly explored in further works.

As described above, anemia was shown to contribute to decreasing overall survival in different types of cancer (23, 25), including GBM (16–19), probably due to increased tumor aggressiveness. The exact mechanisms, though, are mostly unknown. Nevertheless, hypoxia is speculated to play a significant role (40) since it is recognized that hypoxic GBM environment attributes much to tumor progression and reduces radiotherapy therapeutic effect (41). It is possible that anemia can force hypoxic effect in GBM, potentiate pseudopalisading cells (i.e., by increasing matrix metalloproteinases activity) (42) and thus, propagate tumor invasiveness that will eventually result in a poorer prognosis of anemic patients.

It is well-known that a decrease in the level of hemoglobin and the resulting decrease in oxygen delivery in healthy tissues lead to disruption or even death of the cells that form these tissues. In cells of malignant tumors, including GBM, hypoxia caused by a decrease in hemoglobin, on the contrary, leads not to death, but supports rapid proliferation of these cells through various mitochondrial and biochemical mechanisms by: free radicals formation affecting nucleotides formations, increasing HIF-1α (hypoxia-inducible factor-1α), VEGF signaling pathway increase and more (43–45). Anemia in GBM patients, therefore, can further potentiate hypoxic stress and shorten survival even more, as posited from the results presented in this work.

Kleinberg et al. (46) conducted phase II clinical trial results of GBM patients treated with RSR13, a radiation sensitizer, before radiation therapy, which has been shown to enhance oxygen tissue delivery and showed a tendency to improve survival. Phase III of this trial has not been conducted so far. Interestingly, low testosterone level is related to low hemoglobin values in elderly males (mean age 73–75) and testosterone supplementation given to mildly anemic males in a randomized controlled trial recently conducted (47), was shown to correct it. In light of the results presented here, and the literature review, it may be relevant to re-evaluate possibilities to supplement the current GBM treatment paradigm with other modalities, in an attempt to elevate hemoglobin level or reduce RDW value when necessary. As an example, prospective pre-operative evaluation of testosterone level in GBM male patients' and its correction in cases of low hemoglobin levels could improve overall survival. Re-evaluation of other available treatments, i.e., RSR13 or other compounds, could also be beneficial to anemic GBM patients' prognosis.

This study cannot determine the exact mechanism for anemia in GBM patients and cannot exclude the possibility that the tumor itself caused reduced hemoglobin levels or increase RDW. GBM is known to induce severe hematological disturbances, i.e., increase coagulopathy and DVT risk (48, 49). According to the results presented here, it is less likely that GBM induces low hemoglobin level by bone marrow suppression followed by reduced RBCs production rate, since PLT and WBC counts were not decreased. Regardless of the causes of anemia or its timing with GBM diagnosis, it caused a negative effect on patients' overall survival.

In the presented study, normal hemoglobin level separately and in combination with RDW < 14% unraveled a group of GBM patients that could benefit greatly from these parameters integrated into their personalized patient profile. Moreover, in light of the findings mentioned above, it may be worthwhile to review pre-operative hemoglobin and RDW values as additional prognostic screening parameters and to include therapies to enhance hemoglobin level in the future as part of an accepted GBM treatment paradigm.

The statistical study limitations are mostly well-defined since it is a retrospective study of a relatively small number of patients due to the rarity of the malignancy and data collected from a single institute. The main concern is the probability of false-positive findings due to the multiple retrospective hypothesis testing. In the present study, this effect was managed using the BH false discovery rate adjustment (35)—still not a usual practice, as it should be in these types of studies.

The analysis given in this study raised several important unanswered questions that should be addressed in future research. For example, to identify the molecular features that characterize normal hemoglobin and low RDW group of GBM patients in an attempt to shed light on the mechanism responsible for their favorable prognosis. Additionally, it will be imperative to validate these results in a larger patients' group and to evaluate the contribution of these parameters correction to GBM patient's prognosis prospectively.

## Data Availability Statement

The datasets generated for this study are available on request to the corresponding authors.

## Ethics Statement

This study involving human participants was conducted in accordance with the ethical standards of Soroka University Medical Center research committee (institutional approval number: SOR 0170-15) and with the 1964 Helsinki declaration and its later amendments or comparable ethical standards. Due to the retrospective nature of this study and the unfortunate limited lifespan of patients enrolled in this study, informed consent was not possible to obtain and was waived by the institutional ethical committee.

## Author's Note

Glioblastoma multiforme is an incurable disease. Besides the well-established prognostic factors such as younger age, KPS>70, and tumor resection supplemented with oncological treatment, other factors, including RBCs parameters linked to prognosis, remain inconclusive so far. In this study, a comprehensive retrospective inspection of RBCs features of 170 GBM patients highlighted a subgroup of patients with enhanced overall survival that were treated with resection and oncological therapy and was additionally characterized by age under 70 with normal hemoglobin level and RDW < 14%. The beneficial median overall survival of this subgroup was measured as 21.1 months, similar to TTFields treatment applied nowadays in addition to the established chemo-radiation standard first-line treatment regimen. According to the data presented in this paper, normalization of hemoglobin levels, and RDW prior to surgical intervention may improve GBM patient's prognosis.

## Author Contributions

TK-E, YEl, and IM: study conception, data collection, data analysis, manuscript writing, editing, and final approval of manuscript. VM, LD, AN, MA-V, RB, ST, AA, TZ, YEs, KL, YK, and VD: data collection, data analysis, manuscript editing, and final approval of manuscript.

## Conflict of Interest

The authors declare that the research was conducted in the absence of any commercial or financial relationships that could be construed as a potential conflict of interest.
